# AI‐Based Wound Measurement in a Telemedicine‐Style Workflow: Agreement With In‐Person Reference Measurements Compared With Individual Physicians and Expert Consensus

**DOI:** 10.1111/iwj.71043

**Published:** 2026-10-15

**Authors:** Tassilo Dege, Vanessa Borst, Luisa Deutzmann, Jan Hendrik Maiwald, Samuel Kounev, Astrid Schmieder

**Affiliations:** ^1^ Department of Dermatology, Venereology and Allergology University Hospital Würzburg Würzburg Germany; ^2^ Department of Computer Science University of Würzburg Würzburg Germany

**Keywords:** artificial intelligence, chronic wounds, study, telemedicine, wound care

## Abstract

Accurate wound size measurement is essential for monitoring healing, yet visual estimates are variable—especially in telemedicine‐style assessments. To compare AI‐based wound size estimates with physician image‐based estimates using in‐person measurements as ground truth (GT). In a prospective dataset of bedside wound photographs (Dec 2024–Apr 2025), GT height and width were measured in person by two experienced raters using a calibrated ruler. A set of ArUco markers placed adjacent to the wound at the same skin level enabled image‐based retrieval of real‐world size information. Five physicians estimated height and width from images in a telemedicine‐style workflow. Two deep‐learning segmentation architectures (TransNeXt‐Tiny [TransNeXt] and VWFormerConvNeXtS [VW‐Conv]) generated automated size estimates. Agreement and error were quantified using ICC(2,1), Pearson's *r*, MAE, bias and Bland–Altman limits of agreement. In addition, pixel‐level segmentation performance of both models was quantified against expert‐annotated reference masks using Intersection‐over‐Union (IoU), the Dice coefficient, precision and recall, reported as macro‐averaged (per‐image) and micro‐averaged (pixel‐pooled) values. AI achieved excellent agreement for height (TransNeXt ICC = 0.96, MAE = 0.82 cm; VW‐Conv ICC = 0.95, MAE = 0.68 cm) with minimal bias. For width, AI showed moderate‐to‐strong agreement with a tendency to underestimation (TransNeXt ICC = 0.86, MAE = 0.69 cm, bias = −0.42 cm; VW‐Conv ICC = 0.77, MAE = 0.74 cm, bias = −0.64 cm). Individual physician performance was heterogeneous, while a human consensus improved agreement (height ICC = 0.94, MAE = 0.82 cm; width ICC = 0.91, MAE = 0.65 cm). At the pixel level, TransNeXt reached a macro‐averaged Dice of 0.886 (IoU 0.805) and VW‐Conv a macro‐averaged Dice of 0.818 (IoU 0.733), with TransNeXt showing the more consistent per‐image performance. AI‐based wound measurement was more consistent than single physician estimates and reached agreement comparable to, but not exceeding, a consensus of experts. Within the constraints of this single‐centre validation study, these findings support AI‐assisted measurement as an objective aid for standardised wound monitoring in telemedicine and clinical research; confirmation in larger, multicentre and prospectively recruited cohorts is required.

## Key Points

- AI demonstrated excellent agreement for image‐based wound measurements when compared to in‐person ground truth, with pixel‐level segmentation accuracy (Dice 0.886, IoU 0.805 for TransNeXt) in the range reported for real‐world clinical wound imaging algorithms.
- Physician image‐based estimates were heterogeneous, while an expert consensus improved agreement, highlighting the variability of single‐rater assessments and the value of consensus for robust wound size estimation in remote workflows.
- An explainability analysis supported clinical plausibility, with models focusing on wound regions even in challenging cases (e.g., irregular borders, erythema, atypical locations, tattoos).
- Practical suggestion: Combining standardised wound photography with AI‐assisted measurement can reduce variability and improve comparability of wound dimensions across clinicians, centres and follow‐up visits—particularly in telemedicine and other decentralised care pathways.

## 1 Introduction

Accurate and reproducible wound size measurement is a cornerstone of wound care, providing an objective basis for monitoring healing, guiding therapeutic decisions and assessing treatment efficacy. Traditionally, wound dimensions are obtained through direct measurement or manual tracing, while in many clinical settings, healthcare professionals often rely solely on visual estimation due to time constraints. However, human assessment is prone to bias and considerable inter‐ and intra‐observer variability, particularly when wounds are irregularly shaped, located on contoured surfaces or imaged under suboptimal conditions. Accordingly, a study of wound photographs found AI‐generated tracings showed error patterns largely comparable to wound experts, while highlighting that clinician agreement on wound characteristics may vary substantially across reviewers [1]. Recent advances in artificial intelligence (AI) and computer vision have opened new possibilities for automated wound assessment [2]. Deep learning–based segmentation and measurement tools have demonstrated promising accuracy and reproducibility in both experimental and clinical settings. By leveraging large datasets and standardised measurement algorithms, AI has the potential to overcome limitations of human subjectivity, providing consistent, scalable and rapid wound size estimates. Beyond measurement, large‐scale machine learning models have also shown high early predictive performance for 12‐week wound healing outcomes, highlighting the potential of AI to support timely identification of patients who may benefit from advanced (and costly) treatment strategies [3]. Nonetheless, the clinical adoption of such systems requires robust validation against established reference standards and direct comparison with human performance, ideally also demonstrating added value in ‘human‐with‐machine’ workflows—for example, in a prospective JAMA Dermatology study, dermatologists significantly improved sensitivity, specificity and overall accuracy (and reduced unnecessary excisions) when re‐evaluating lesions with support from a market‐approved CNN [4].

Several recent studies have addressed these challenges. Chan et al. clinically validated an AI‐enabled wound imaging mobile application, demonstrating high agreement with manual planimetry across a variety of wound types [5]. Similarly, Carrión et al. used deep learning–based segmentation to extract wound size and morphology from photographic datasets, highlighting the potential of AI tools to standardise wound measurement in research and clinical practice [6]. While these investigations confirm the feasibility of AI‐based wound assessment, few have systematically compared AI predictions directly with human estimations across a consistent dataset in real‐world conditions. More recently, Dabas et al. applied a colour‐space‐based image‐processing algorithm to pressure injury photographs acquired by nurses during routine clinical practice, reporting an Intersection‐over‐Union of up to 0.85 and demonstrating that automated surface‐area extraction is feasible under real‐world documentation conditions [7]. Li et al. combined K‐means clustering with a printed reference marker to compensate for variable camera distance in outpatient wound photography, [8] while a recent scoping review of three‐dimensional wound assessment technologies concluded that, despite improved geometric precision, clinical validation against established reference standards and evidence of prognostic utility remain limited [9]. In parallel, technical work has established Dice‐ and IoU‐based benchmarks for deep‐learning wound segmentation, but these are rarely linked to centimetre‐level measurements obtained by clinicians at the bedside [10].

The overarching goal of this project was to create and validate an explainable AI‐based wound size estimation tool that can be readily used by the medical community. The present study therefore aims to directly compare AI‐predicted wound sizes with human‐predicted wound sizes, using manually annotated measurements as the ground truth. We assess agreement, bias and variability between the two approaches, quantify interrater differences among human assessors and evaluate the potential of AI to serve as a reliable and objective alternative for wound measurement in diverse clinical scenarios.

## 2 Material and Methods

### 2.1 Ethical Consideration

This analysis was conducted in accordance with the Declaration of Helsinki and ethical approval was obtained from the local ethics committee (censored and censored). Written informed consent was obtained from all participating patients prior to image acquisition and data use.

### 2.2 Study Design and Dataset Creation

We performed a prospective analysis of a curated dataset of wound images collected at a university hospital between December 2024 and April 2025. In total, 40 wound images were acquired; after applying the predefined inclusion and exclusion criteria, 20 images were retained for the final analysis. Images included various wound etiologies and were obtained using standardised photographic protocols, with a known reference object (supplemental material) included in the field of view. The reference object comprises four ArUco markers to facilitate detection within the image and a Macbeth colour chart containing 24 colour patches that could be used for colour calibration in the future (Supporting Information).

Inclusion criteria were as follows:

- High‐resolution colour images with a visible wound margin and reference object.
- Availability of corresponding ground truth wound measurements.

Exclusion criteria were

- Images with severe artefacts, missing reference scale or incomplete wound visibility.
- Wounds obscured by dressings or debris.

### 2.3 Wound Size Assessment and Prediction

Ground truth (GT) wound sizes were established by two independent raters with ≥ 5 years of experience in wound assessment. At the time of image acquisition, each rater measured wounds in person using a calibrated ruler placed directly on the patient's skin to determine the maximum length (height) and maximum width. In addition, an ArUco reference marker was placed adjacent to the wound at the same skin level to provide a standardised reference for subsequent image‐based analysis. If discrepancies between the two measurements occurred, the raters reviewed the values and agreed on a consensus measurement, which was used as the final GT height and width. These in‐person measurements served as the reference standard for all subsequent analyses. GT raters were blinded to both the automated AI predictions and the human image‐based assessments. This structured, Delphi‐style consensus procedure was defined a priori; individual pre‐consensus values were not retained so that only the agreed consensus measurement is available as the reference standard.

Image‐based wound size estimations were obtained from five independent physicians (four board‐certified dermatologists and one internal medicine specialist), all with clinical experience in wound assessment. Raters used a custom Python‐based software tool specifically developed for image‐based wound measurement (Figure S1). The tool allowed raters to load wound images and view them alongside the embedded reference object (ArUco marker). Raters then entered their estimated wound height and width into a text box. They could additionally record the estimated percentages of fibrin, necrosis, epithelialization and granulation; however, these values were not included in the present analysis. The tool implements the standard clinical ruler convention, namely the longest wound extent (height) and the longest extent perpendicular to it (width). Raters therefore performed on screen the same measurement operation that they routinely perform at the bedside with a ruler and the identical convention was applied to the AI‐derived contours (Section 2.4.2), so that human estimates, AI predictions and the in‐person reference standard are all based on the same geometric definition and are directly comparable.

This image‐based rating setup was chosen to simulate a *telemedicine*‐style evaluation, where wound size serves as a key indicator of wound status and may reflect clinical improvement or deterioration over time. All raters received standardised instructions and a brief training session with the software prior to data collection. Human assessments were performed while blinded to the GT measurements and the AI predictions. Figure 1 provides an overview of the study workflow and rating procedure.

**FIGURE 1:**
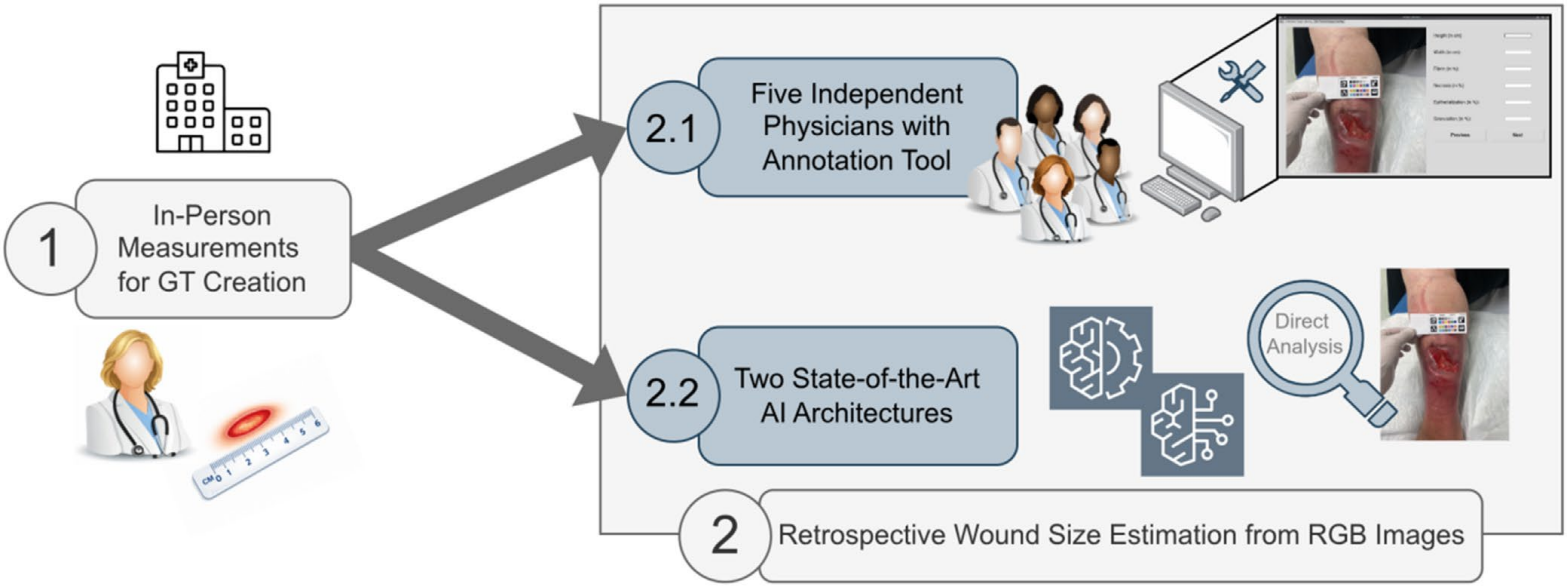
Study workflow for wound size validation. GT wound height and width were measured in person at image acquisition using a calibrated ruler; a set of ArUco markers was placed adjacent to the wound at the same skin level for image analysis. Wound size was then estimated from RGB images by five physicians (telemedicine‐style assessment) and by two AI models (TransNeXt, VW‐Conv) and compared against GT.

### 2.4 AI‐Based Prediction

In this study, we implemented automated wound size estimation using two state‐of‐the‐art deep learning architectures for image segmentation: TransNeXt‐Tiny (TransNeXt) and VWFormer with ConvNeXt‐S backbone (VW‐Conv). These models were selected based on our previous findings [11] which demonstrated their effectiveness in wound segmentation^3^. Both architectures were trained and validated on publicly available datasets of foot ulcer photographs (*n* = 2887), following a standardised pipeline that included image preprocessing, augmentation and five‐fold cross‐validation. To assess their robustness to new data, we evaluated the models on a custom dataset of expert‐annotated wound images (*n* = 343) collected at our university hospital. This dataset was used to simulate real‐world scenarios and evaluate the models' performance outside of the training environment. The underlying benchmarking study comparing 12 deep learning architectures for wound segmentation has been published in a technical paper presented at a major international computer science conference (censored). The trained models, along with the implementation, are available as open‐source software via the a repository (censored), demonstrating the feasibility and reusability of this approach for the medical community.

#### 2.4.1 Model Selection and Ensemble Approach

Two models from the preceding benchmark study were selected for further analysis: TransNeXt, which demonstrated the strongest overall segmentation performance and robustness to previously unseen data and VW‐Conv, which achieved the highest expert choice rate. The expert choice rate was derived from dermatologist assessments in which the predicted segmentation masks of multiple models were manually compared.

For each of the two selected model architectures, five independently trained sub‐models were used, with each sub‐model trained on a different data split. Each sub‐model generated a binary segmentation by assigning each pixel to either the wound (1) or background class (0). For binary mask generation, the model logits were thresholded at 0.5, corresponding to a probability threshold of approximately 0.6225 under the sigmoid transformation. The resulting binary masks from the five sub‐models were combined using pixel‐wise majority voting, with a pixel classified as wound when at least three of the five sub‐models predicted the wound class.

The five sub‐models were treated as independent model realizations, analogous to multiple raters providing predictions for the same image. Majority voting therefore produced a consensus segmentation across the model ensemble, reducing the influence of predictions specific to individual training splits (Figure 2).

**FIGURE 2:**
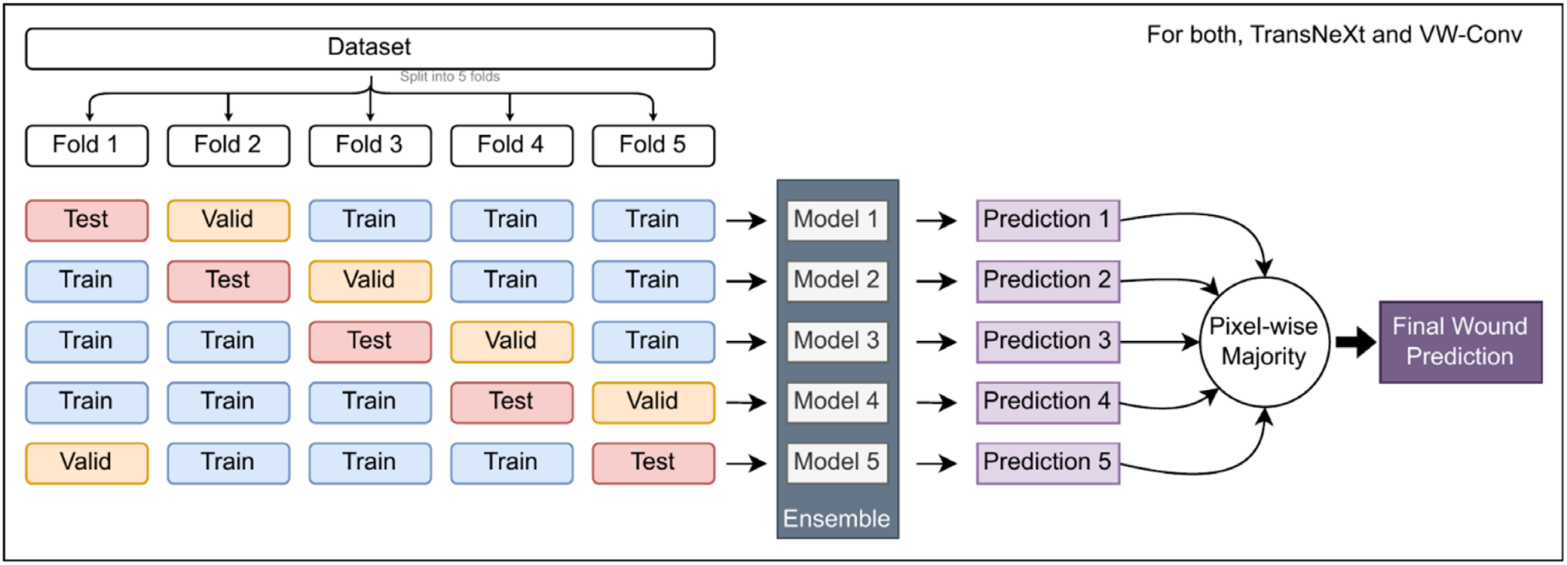
Ensemble technique for robust size estimation. The dataset was split into five folds and five models were trained on slightly different data subsets. For each image, all models produce a wound mask and the final mask is obtained by pixel‐wise majority vote. This was applied to both TransNeXt and VW‐Conv to improve robustness.

#### 2.4.2 Conversion to Physical Wound Dimensions

For each image, the wound's height and width are determined based on the AI‐generated pixel‐level segmentation, following standard clinical measurement procedures. The *longest diagonal of the segmented wound contour* is identified and designated as the *height*, while the *longest perpendicular line* to this diagonal is used to determine the *width*. These measurements, initially in pixels, are then converted into physical dimensions using the same reference object placed alongside the wound as in the human assessments. This conversion enables the AI system to provide wound dimensions estimates that are directly comparable to clinician measurements.

The AI‐based segmentation and size estimation pipeline was integrated into a custom Python‐based application, which outputs the predicted wound size for both height and width separately. This enables a direct comparison of the AI‐predicted wound measurements with clinician estimates and in‐person ground truth, all within a single workflow.

#### 2.4.3 Evaluation of Pixel‐Level Segmentation Performance

To allow direct comparison with previously published segmentation studies, the predicted wound masks of both ensemble models were additionally evaluated against pixel‐level reference masks. Reference masks were annotated for all 20 study images by an experienced dermatologist using the CVAT annotation platform. These annotation masks are conceptually distinct from the measurement reference standard: the in‐person ruler measurement served as the reference for wound dimensions, whereas the manual masks served as the reference for wound delineation.

For each image, a pixel‐wise confusion matrix (true positives, false positives, false negatives, true negatives) was computed between the binarised reference mask and the model prediction, from which IoU, precision, recall and the Dice coefficient were derived. Two levels of aggregation are reported. The macro‐averaged score is the unweighted mean of the per‐image metrics and gives every wound equal weight irrespective of its size. The micro‐averaged (globally pooled) score is obtained by first summing the confusion‐matrix counts across all images and deriving the metrics from the pooled totals, which gives every pixel equal weight and therefore weights large wounds more strongly. Reporting both levels separates per‐image consistency (macro) from overall pixel‐level agreement (micro); the divergence between them is informative about size‐dependent segmentation behaviour. Because wounds occupy only a small fraction of each photograph, pixel‐level accuracy is dominated by correctly classified background pixels. It is therefore reported for completeness only, is not informative under this degree of class imbalance and is not interpreted further. Per‐image metrics, together with technical details of mask decoding and of the EXIF‐orientation alignment applied between predictions and annotations, are provided in the Supporting Information.

### 2.5 Statistical Analysis

All statistical analyses were performed in Python (v3.13) using pandas for data handling, NumPy for numerical computations and matplotlib for visualization. Agreement between AI predictions, human estimates and ground truth (GT) measurements was assessed using the Intraclass Correlation Coefficient (ICC; two‐way random‐effects, absolute agreement; ICC(2,1)), which quantifies how closely two measurement methods agree on an absolute scale (values closer to 1 indicate stronger agreement). Linear association was additionally summarised using Pearson's correlation coefficient (*r*).

Prediction error was quantified using the mean absolute error (MAE), representing the average absolute deviation from GT in centimetres. Systematic differences were assessed by the mean difference (bias), defined as (method−GT), where negative values indicate underestimation relative to GT. To characterise variability across the measurement range, we used Bland–Altman analysis, which plots the differences against the mean of the two methods and reports 95% limits of agreement (LoA = bias ±1.96 × SD of differences). Bland–Altman plots were chosen because they evaluate agreement and potential proportional bias rather than correlation alone. Human assessor variability was characterised by computing rater‐specific agreement with GT (ICC(2,1), Pearson's *r*, MAE, bias and LoA) and by summarising human performance using (i) a mean‐rater approach (averaging rater‐specific metrics) and (ii) a consensus estimate (mean across raters per image) compared against GT. Where applicable, subgroup analyses explored differences by wound size, wound type and image quality.

## 3 Results

### 3.1 Dataset and Wound Characteristics

Ground truth (GT) wound size was defined as the product of the longest and the widest in‐person measured diameter. Median GT wound area (calculated by height × width) was 18.2 cm^2^ (IQR 4.1–29.0 cm^2^; range 1.1–105.0 cm^2^). Nine wounds (45.0%) were smaller than 10 cm^2^, 6 (30.0%) were between 10 cm^2^ and 30 cm^2^ and 5 (25.0%) were greater than 30 cm^2^. For image‐based human assessment, five physicians (four dermatologists, one internist) provided wound measurements for 20 images.

### 3.2 Pixel‐Level Segmentation Performance

Across the 20 images, TransNeXt achieved a macro‐averaged IoU of 0.805 and Dice of 0.886 (precision 0.894, recall 0.901) and a micro‐averaged IoU of 0.822 and Dice of 0.902 (precision 0.932, recall 0.874). VW‐Conv achieved a macro‐averaged IoU of 0.733 and Dice of 0.818 (precision 0.879, recall 0.847) and a micro‐averaged IoU of 0.724 and Dice of 0.840 (precision 0.921, recall 0.773) (Table 1).

**TABLE 1:** Pixel‐level segmentation performance of both ensemble models against expert‐annotated reference masks, reported as macro‐averaged (unweighted mean of per‐image metrics) and micro‐averaged (confusion‐matrix counts pooled across all images) values.

Model	Aggregation	IoU	Dice	Precision	Recall	Accuracy^a^
TransNeXt	Macro (per image)	0.805	0.886	0.894	0.901	0.995
TransNeXt	Micro (pooled)	0.822	0.902	0.932	0.874	0.995
VW‐Conv	Macro (per image)	0.733	0.818	0.879	0.847	0.993
VW‐Conv	Micro (pooled)	0.724	0.840	0.921	0.773	0.992

Abbreviations: IoU, Intersection‐over‐Union; VW‐Conv, VWFormerConvNeXtS.

^a^ Pixel‐level accuracy is dominated by correctly classified background pixels and is reported for completeness only; it is not informative under this degree of class imbalance.

Per‐image performance was more consistent for TransNeXt (Dice 0.886 ± 0.089; median 0.903, IQR 0.844–0.954, range 0.682–0.984) than for VW‐Conv (Dice 0.818 ± 0.213; median 0.911, IQR 0.795–0.943, range 0.171–0.978). The median Dice of VW‐Conv was thus marginally higher, whereas its mean was reduced by two outlier images with markedly incomplete segmentation (Dice 0.17 and 0.38). In a paired comparison, the difference in Dice between the two architectures did not reach statistical significance (Wilcoxon signed‐rank test, *p* = 0.11); the models are therefore comparable in central tendency but differ in robustness, with TransNeXt showing fewer failure cases.

For both models, pooled precision exceeded pooled recall (TransNeXt 0.932 vs. 0.874; VW‐Conv 0.921 vs. 0.773), whereas per‐image precision and recall were approximately balanced. This pattern indicates net under‐segmentation concentrated in the larger wounds and provides a mechanistic explanation for the negative width bias observed in the measurement analysis (Section 3.3). Pixel‐level accuracy exceeded 0.99 for both models but reflects the predominance of background pixels rather than segmentation quality and is not interpreted further.

### 3.3 AI Performance Versus Ground Truth

In all study images, the AI pipeline successfully generated clinically plausible wound masks and metric wound sizes, including challenging cases with very large ulcers, irregular shapes and tattoos (Figure 4). This demonstrates the technical feasibility of integrating an open‐source deep‐learning framework into routine bedside photography for standardised wound size assessment. The underlying code and trained models are publicly available, enabling independent validation and reuse in other cohorts.

For the first AI model TransNeXt, agreement with GT was ICC = 0.96 for height (*r* = 0.96, MAE = 0.82 cm, bias = +0.02 cm, LoA −2.06 to +2.11 cm) and ICC = 0.86 for width (*r* = 0.93, MAE = 0.69 cm, bias = −0.42 cm, LoA −2.43 to +1.51 cm). For the second AI model VW‐Conv, agreement with GT was ICC = 0.95 for height (*r* = 0.95, MAE = 0.68 cm, bias = −0.16 cm, LoA −2.50 to +2.19 cm) and ICC = 0.77 for width (*r* = 0.86, MAE = 0.74 cm, bias = −0.64 cm, LoA −3.10 to +1.81 cm) (Table 2).

**TABLE 2:** Agreement of wound height and width across TransNeXt, VW‐Conv, mean rater approach and consensus rater approach using ICC, Pearson's *r*, MAE, bias and limits of agreement.

Dimension	Group	ICC	*r*	MAE	Bias	LoA
Height	TransNeXt	0.96	0.96	0.82 cm	+0.02 cm	−2.06 to +2.11 cm
	VW‐Conv	0.95	0.95	0.68 cm	−0.16 cm	−2.50 to +2.19 cm
	Mean rater approach	0.90	0.91	1.01 cm	−0.34 cm	−3.28 to +2.60 cm
	Human consensus (mean)	0.94	0.96	0.82 cm	−0.34 cm	−2.58 to +1.90 cm
Width	TransNeXt	0.86	0.93	0.69 cm	−0.42 cm	−2.43 to +1.51 cm
	VW‐Conv	0.77	0.86	0.74 cm	−0.64 cm	−3.10 to +1.81 cm
	Mean rater approach	0.84	0.86	0.90 cm	−0.30 cm	−2.52 to +1.95 cm
	Human consensus	0.91	0.94	0.65 cm	−0.21 cm	−1.99 to +1.43 cm

Abbreviations: ICC, Intraclass Correlation Coefficient; LoA, limits of agreement; VW‐Conv, VWFormerConvNeXtS.

### 3.4 Human Image‐Based Assessment Versus Ground Truth

For height, agreement with GT varied across raters (ICC 0.80–0.96, *r* 0.85–0.96), with MAE 0.80–1.34 cm and small systematic bias ranging from −1.07 to +0.23 cm (LoA −4.89 to +3.34 cm, rater‐specific). For width, agreement was more heterogeneous (ICC 0.62–0.92, *r* 0.68–0.94), with MAE 0.66–1.12 cm and bias −0.66 to +0.06 cm (LoA −4.01 to +2.68 cm, rater‐specific).

Across all images, we summarised human performance in two ways:

- rater performance (mean), where agreement metrics were computed per rater vs. GT and then averaged across raters; and
- human consensus (mean), where raters' measurements were first combined into one value per image and compared against GT.

Agreement with GT for both AI models and for the mean‐rater and consensus approaches is summarised in Table 2. Using the mean rater approach, agreement with GT was ICC = 0.90 for height (*r* = 0.91; MAE = 1.01 cm; bias = −0.34 cm; LoA −3.28 to +2.60 cm) and ICC = 0.84 for width (*r* = 0.86; MAE = 0.90 cm; bias = −0.30 cm; LoA −2.52 to +1.95 cm).

Using human consensus, agreement improved further: for the mean consensus, height ICC = 0.94 (*r* = 0.96; MAE = 0.82 cm; bias = −0.34 cm; LoA −2.58 to +1.90 cm) and width ICC = 0.91 (*r* = 0.94; MAE = 0.65 cm; bias = −0.21 cm; LoA −1.99 to +1.43 cm).

### 3.5 Direct Comparison of AI and Human Performance

For height, both AI models achieved excellent agreement with GT (ICC 0.95–0.96), comparable to or slightly exceeding the human mean consensus (ICC = 0.94). For width, the human consensus performed best overall (ICC = 0.91; MAE = 0.65 cm), whereas both AI models showed stronger negative bias (greater underestimation; bias −0.42 to −0.64 cm) and lower ICC (0.77–0.86). The corresponding Bland–Altman plots for height and width are shown in Figure 3.

**FIGURE 3:**
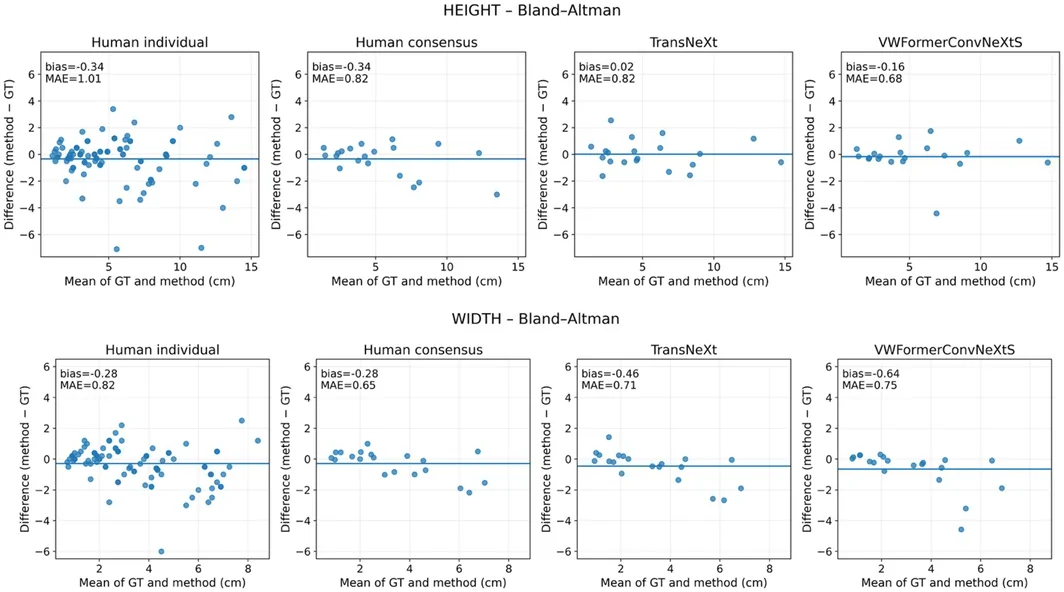
Bland–Altman plots for wound size measurements (height and width). Each point represents one wound image. The *x*‐axis shows the mean of the ground truth (GT) and the respective method and the *y*‐axis shows the difference (method−GT), where negative values indicate underestimation relative to GT. Solid horizontal lines indicate the mean difference (bias). Panels compare individual human ratings, the human consensus estimate (mean across raters) and the AI models (TransNeXt and VWFormerConvNeXtS) against GT.

Overall, both AI models were comparable to the human consensus in height accuracy (similar MAE; smaller absolute bias for TransNeXt), while for width the human consensus showed the best overall agreement (highest ICC and lowest MAE) and both AI models showed stronger negative bias (greater underestimation) than the human consensus.

### 3.6 Explainable AI (XAI)

To qualitatively assess whether the models relied on clinically plausible image regions, we created and analysed Gradient‐weighted Class Activation Mapping (Grad‐CAM) visualizations for all images. All images showed sharp demarcation of surrounding skin even in challenging cases as seen in the representative cases of Figure 4. In ID 1 (foot, deep lesion with visible tendon), the attribution concentrated on the wound core and its sharply demarcated margins, consistent with a depth‐ and edge‐driven decision. In ID 2 (erythematous border), activations could accurately differentiate between wound area and its red inflamed border zone, reflecting the visual importance of the periwound transition in such cases. For ID 3 (abdominal location), the heatmaps primarily highlighted the wound area rather than surrounding background skin texture. In ID 4 (tattoo), attributions remained centred on the index wound (largest wound used for GT and all comparisons) despite the presence of a second visible wound and tattoo patterns as potential visual confounders, indicating relative robustness to these sources of distraction (Figure 4).

**FIGURE 4:**
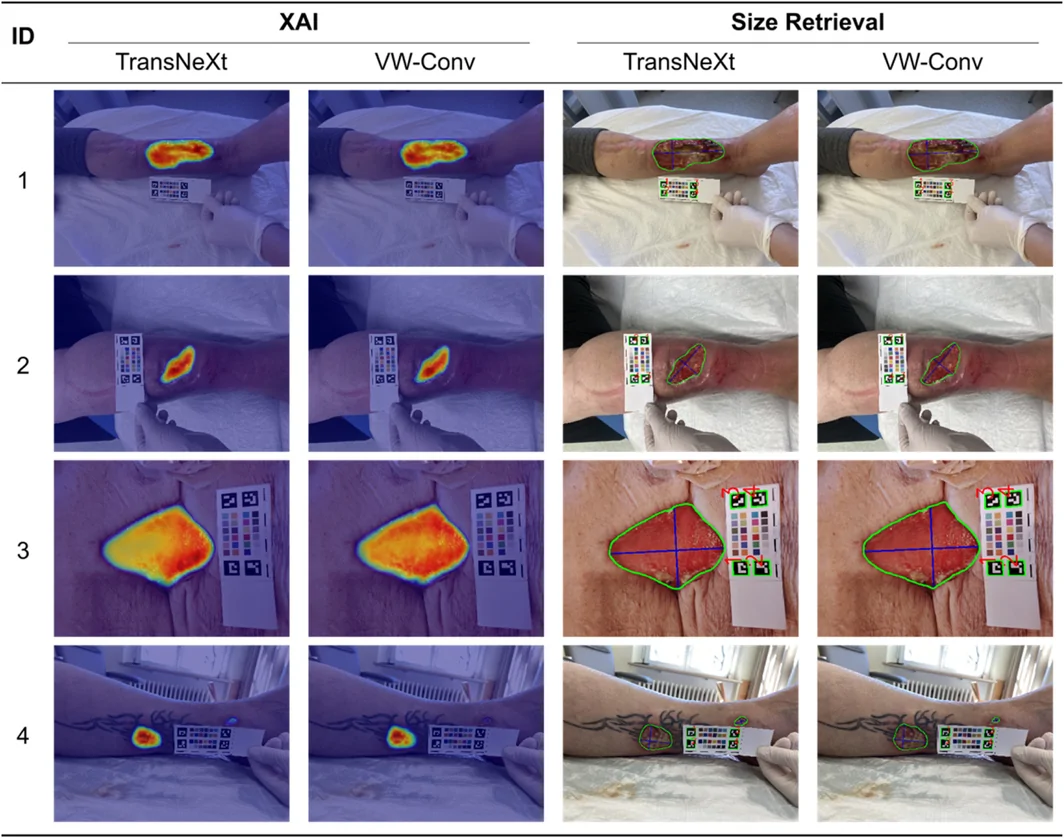
Explainability and automated size retrieval in representative cases. Grad‐CAM heatmaps (left; TransNeXt and VW‐Conv) illustrate image regions driving model predictions and the corresponding automated wound size retrieval outputs (right) show the detected wound contour and measurement axes for the same four example images (IDs 1–4). Examples include challenging scenarios such as deep/irregular ulcers, erythematous wound borders, abdominal location and tattoo patterns (ID 4; index wound shown despite a second visible wound).

## 4 Discussion

Rapid advances in deep learning and artificial intelligence (AI) already have the potential to bring about lasting change in medical care. In the treatment of chronic wounds in particular, the use of e‐health and *telemedicine* solutions is opening up new opportunities to support diagnostic processes, improve the quality of treatment and reduce costs at the same time [12, 13].

In a study by Chan et al., an AI‐supported smartphone application (CARES4WOUNDS, C4W) was developed for wound measurement and showed good inter‐ and intra‐rater reliability compared to traditional methods [5]. However, this work did not directly benchmark AI‐derived measurements against individual physicians and a physician consensus using an in‐person ground truth reference and images were acquired under controlled conditions, limiting transferability to real‐world care—particularly telemedicine‐style image review. Similarly, Stefanelli et al. recently reported a comprehensive methodological framework for building a mobile AI wound assessment tool, including large‐scale multicentre data collection, wound segmentation and tissue analysis and mobile deployment optimization; however, their evaluation primarily focused on segmentation performance (e.g., Dice/IoU) rather than centimetre‐level agreement with clinician estimates against an in‐person reference standard [14]. In addition, implementation‐focused work has highlighted the potential of AI‐driven wound platforms to improve access and workflow efficiency in rural and remote settings, underscoring the need for clinically interpretable validation of automated measurements in real‐world workflows [15]. In another study by Fong et al., wound measurement was predominantly performed on site by specialised wound care professionals rather than as part of a telemedical assessment, limiting transferability to decentralised image acquisition scenarios [16]. Hence, the transferability of these results to telemedical application scenarios with decentralised image acquisition therefore remains limited. Taken together, these gaps motivated the present head‐to‐head agreement analysis of AI and clinicians using a telemedicine‐style image review setup with in‐person ground truth measurements.

Most directly relevant to the present work, Dabas et al. developed a colour‐space‐based image‐processing pipeline for pressure injuries photographed by nurses during routine care and reported an Intersection‐over‐Union of up to 0.85, demonstrating that automated surface‐area extraction is achievable under uncontrolled, real‐world documentation conditions [7]. The deep‐learning ensembles evaluated here achieved a comparable order of segmentation accuracy (TransNeXt IoU 0.805 macro, 0.822 micro) on a heterogeneous set of wound etiologies rather than a single wound type and were, in contrast to that work, benchmarked head‐to‐head against five physicians using an in‐person ruler measurement as the reference standard. Similar considerations apply to marker‐based approaches designed to correct for variable camera distance [8] and to three‐dimensional wound assessment technologies, for which a recent scoping review concluded that clinical validation and prognostic utility remain insufficiently established [9]. Reporting Dice and IoU alongside centimetre‐level agreement therefore positions the present results both against the technical segmentation literature [10] and against clinically oriented implementation studies, which to date have rarely been reported together.

Overall, both the TransNeXt and VW‐Conv models showed excellent agreement with the ground truth for the measurement of wound height and good agreement with the ground truth of wound width (height ICC: TransNeXt 0.96, VW‐Conv 0.95; width ICC: TransNeXt 0.86, VW‐Conv 0.77). The agreement values achieved were comparable overall and, in some cases, slightly higher than those of the human assessment (height ICC: mean rater approach 0.90, consensus rater approach 0.94; width ICC: mean rater approach 0.84). For the measurement of wound width, the human consensus rater approach showed the best agreement with the ground truth (ICC = 0.91).

In an earlier study, we were already able to show that our AI model exhibited a very high generalizability in the segmentation of chronic wounds compared to other architectures. In particular, for out‐of‐distribution (OOD) data, including images collected independently at our university hospital, the model continued to show high segmentation performance, indicating robust generalization across different data sources [11]. Building on this and informed by a prior systematic evaluation of chronic wound apps [17] demonstrating limited overall quality and the need for improved, patient‐centred development, the present measurement pipeline was translated into clinical application and is currently being evaluated prospectively; this is detailed in Section 4.1 (trial registered at the German Clinical Trial Register, DRKS00038408) [18].

Across agreement and error metrics, AI‐based wound measurements surpassed the average individual rater (mean‐rater approach) and were competitive with the clinician consensus, indicating comparable or improved measurement performance relative to human image‐based assessment. Both clinicians and AI tended to slightly underestimate wound dimensions versus ground truth, with the exception of TransNeXt height, which showed negligible positive bias (+0.02 cm).

The segmentation analysis offers a mechanistic explanation for this systematic underestimation of width. For both models, pooled precision exceeded pooled recall while per‐image precision and recall were balanced, indicating that missed wound pixels accumulate disproportionately in the largest wounds. Because width is derived as the longest extent perpendicular to the principal axis, a conservative delineation of the wound margin translates more directly into an underestimated width than into an underestimated height, which is determined by the longest overall extent and is comparatively robust to marginal pixel loss. The binarisation operating point used for mask generation (Section 2.4.1) is comparatively conservative and is likely to contribute to this behaviour, since a higher effective probability threshold preferentially removes low‐confidence pixels, which are concentrated at the wound margin. This is consistent with the negligible height bias observed for TransNeXt (+0.02 cm) alongside a width bias of −0.42 cm and it identifies margin delineation in large, irregular wounds as a concrete target for further model refinement. Calibration of the binarisation threshold therefore represents a further and readily accessible target for refinement alongside margin delineation itself.

Explainable AI (XAI) techniques such as Gradient‐weighted Class Activation Mapping (Grad‐CAM) have been widely adopted in medical imaging visualizations [19]. Grad‐CAM showed that both TransNeXt and VW‐Conv consistently considered the relevant wound areas. This was also the case under atypical conditions, including exposed tendons, erythematous wound edges, atypical locations and ulcerations in the area of tattooed skin (Figure 4). These findings indicate robust detection of the relevant wound areas and good generalizability of the models across different clinical presentations.

### 4.1 Clinical Implications

For clinical practice, the relevant comparison is not whether AI outperforms an expert panel, but whether it can replace the single, unaided visual estimate made in most routine and telemedical encounters. Individual physician agreement with the in‐person reference varied substantially in this study (height ICC 0.80–0.96; width ICC 0.62–0.92), whereas the automated pipeline produced a deterministic result for every image, without inter‐observer variability and without additional time at the bedside beyond photographing the wound with the reference marker in the field of view. In longitudinal monitoring, where the clinically meaningful quantity is the change in wound size between visits, a reproducible measurement with a small systematic offset is preferable to an unbiased but highly variable one, because a constant offset largely cancels when successive measurements are compared. The measurement pipeline has accordingly been implemented in the censored application and is currently undergoing prospective clinical evaluation, in which usability, time expenditure and the effect on documentation and treatment decisions in routine care are assessed. The present analysis establishes the measurement validity that is a precondition for that evaluation; demonstration of workflow impact itself remains the subject of the ongoing prospective study.

### 4.2 Limitations

Several limitations must be acknowledged. First, this analysis is based on a small, single‐centre sample of 20 wound images, which limits the precision of the reported agreement estimates and their generalisability to other settings, imaging devices and patient populations; confidence intervals around ICC values derived from a sample of this size are correspondingly wide and performance under less controlled conditions may be lower than reported here. The sample size was constrained by the design of the reference standard, since agreement was assessed against in‐person ruler measurements obtained at the bedside at the moment of image acquisition, which cannot be reconstructed retrospectively and therefore cannot be drawn from existing image repositories. Second, the reference measurements were established by two experienced raters using a predefined, Delphi‐style consensus procedure; individual pre‐consensus values were not retained, so the inter‐rater variability of the reference standard itself could not be quantified. Third, the pixel‐level reference masks were annotated by a single experienced dermatologist, so no inter‐annotator ceiling for segmentation performance is available. In this context, the binarisation threshold applied to the model outputs (Section 2.4.1) corresponds to a probability of approximately 0.62 and is therefore more conservative than the conventional value of 0.5; no threshold sensitivity analysis was performed, so the reported segmentation metrics and the negative width bias should be interpreted as applying to this specific operating point. Fourth, the human comparator was an unaided visual estimate from a photograph, chosen to reflect telemedical practice; the study does not compare the AI pipeline against acetate tracing or digital planimetry and the results should not be read as a claim of superiority over these more elaborate manual methods. Fifth, wound depth, undermining and tissue composition were not assessed and the analysis is restricted to the two linear dimensions in routine clinical use. Finally, the present study establishes measurement validity but does not demonstrate an effect on clinical workflow, documentation quality or patient outcomes; these endpoints are the subject of the ongoing prospective evaluation. The findings should accordingly be regarded as a single‐centre validation requiring confirmation in larger, multicentre and prospectively recruited cohorts.

## 5 Conclusion

In this prospective, telemedicine‐style validation using in‐person ruler measurements as reference standard, AI‐based wound measurement was more consistent than individual physician estimates and approached expert consensus and its pixel‐level segmentation accuracy was in the range reported for real‐world clinical wound imaging algorithms. Within the limits of a single‐centre sample of 20 images, these findings support AI‐assisted measurement as a promising objective aid for standardised wound monitoring in routine care, remote monitoring scenarios and clinical research, rather than as a replacement for expert assessment; confirmation in larger, multicentre and prospectively recruited cohorts is required. The ensemble (‘virtual rater’) design provided consensus‐like predictions and Grad‐CAM suggested clinically plausible focus on wound regions even in challenging cases.

## Funding

This work was supported by the Interdisziplinäres Zentrum für Klinische Forschung (IZKF) (CSP‐25).

## Ethics Statement

This analysis was conducted in accordance with the Declaration of Helsinki and ethical approval was obtained from the local Ethics Committee of the University of Würzburg (20220714 01 and 72/24).

## Consent

Written informed consent was obtained from all participating patients prior to image acquisition and data use and by all physicians who participated in this study.

## Conflicts of Interest

The authors declare no conflicts of interest.

## Supporting information


**Figure S1:** Representative wound images with the expert‐annotated reference contour (green) and the TransNeXt ensemble prediction (magenta) overlaid. Each image was acquired with the reference object (ArUco markers and colour chart) in the field of view for metric calibration. (A, B) Lower‐leg ulcer with fibrinous and granulating areas (A) and large granulating wound (B); in both cases reference and prediction show near‐complete overlap along the entire margin, in (A) despite pronounced periwound erythema. (C) Superficial, largely epithelialising wound in the healing phase; the low contrast between wound bed and adjacent skin makes margin delineation more difficult and the prediction is confined to the central area while the expert annotation includes the surrounding healing zone. (D) Plantar forefoot ulcer; the contour follows the reference closely, while a small additional predicted region over the neighbouring toe reflects confusion with a dystrophic nail.

**Data S1:** Supporting Information.

## Data Availability

The artificial intelligence and software that support the findings of this study are openly available in GitHub at https://github.com/VanessaBorst/woundambit. The images and other data are available from the corresponding author on reasonable request.

## References

[1] R. S. Howell , H. H. Liu , A. A. Khan , et al., “Development of a Method for Clinical Evaluation of Artificial Intelligence–Based Digital Wound Assessment Tools,” JAMA Network Open 4, no. 5 (2021): e217234. 10.1001/jamanetworkopen.2021.7234 PMC8134996 34009348

[2] C. H. Annu and S. Choprac , “Role of Artificial Intelligence in Wound Care: A Narrative Review,” Biomedical Engineering Communications 4, no. 2 (2025): 11.

[3] A. C. Dallmann , M. Sheridan , S. Mattke , and W. Ennis , “Prediction of Healing Trajectory of Chronic Wounds Using a Machine Learning Approach,” Advances in Wound Care 14, no. 12 (2025): 645–654. 10.1089/wound.2024.0095 39508072

[4] J. K. Winkler , A. Blum , K. Kommoss , et al., “Assessment of Diagnostic Performance of Dermatologists Cooperating With a Convolutional Neural Network in a Prospective Clinical Study: Human With Machine,” JAMA Dermatology 159, no. 6 (2023): 621–627. 10.1001/jamadermatol.2023.0905 PMC10157508 37133847

[5] K. S. Chan , Y. M. Chan , A. H. M. Tan , et al., “Clinical Validation of an Artificial Intelligence‐Enabled Wound Imaging Mobile Application in Diabetic Foot Ulcers,” International Wound Journal 19, no. 1 (2022): 114–124. 10.1111/iwj.13603 PMC8684856 33942998

[6] H. Carrión , M. Jafari , M. D. Bagood , H.‐y. Yang , R. R. Isseroff , and M. Gomez , “Automatic Wound Detection and Size Estimation Using Deep Learning Algorithms,” PLoS Computational Biology 18, no. 3 (2022): e1009852. 10.1371/journal.pcbi.1009852 PMC8942216 35275923

[7] M. Dabas , S. Kapp , and A. Gefen , “Utilizing Image Processing Techniques for Wound Management and Evaluation in Clinical Practice: Establishing the Feasibility of Implementing Artificial Intelligence in Routine Wound Care,” Advances in Skin & Wound Care 38, no. 1 (2025): 31–39. 10.1097/ASW.0000000000000246 39836554

[8] K. C. Li , Y. H. Lee , and Y. H. Lin , “Automated Image‐Based Wound Area Assessment in Outpatient Clinics Using Computer‐Aided Methods: A Development and Validation Study,” Medicina 61, no. 6 (2025): 1099. 10.3390/medicina61061099 PMC12194858 40572787

[9] A. A. Mehl , N. Abdullah , P. K. Hembecker , and M. A. de Souza , “Advancing Wound Care With 3‐D Imaging: Clinical Applications, Performance and Future Directions,” Wound Repair and Regeneration 33, no. 5 (2025): e70089. 10.1111/wrr.70089 40917036

[10] G. Scebba , J. Zhang , S. Catanzaro , et al., “Detect‐and‐Segment: A Deep Learning Approach to Automate Wound Image Segmentation,” Informatics in Medicine Unlocked 29 (2022): 100884.

[11] V. Borst , T. Dittus , T. Dege , A. Schmieder , and S. Kounev , “WoundAmbit: Bridging State‐of‐the‐Art Semantic Segmentation and Real‐World Wound Care,” In Machine Learning and Knowledge Discovery in Databases Applied Data Science Track, edited by I. Dutra , M. Pechenizkiy , P. Cortez , et al., (Springer Nature, 2026), 285–303.

[12] N. Ohura , R. Mitsuno , M. Sakisaka , et al., “Convolutional Neural Networks for Wound Detection: The Role of Artificial Intelligence in Wound Care,” Journal of Wound Care 28, no. Sup10 (2019): S13–S24. 10.12968/jowc.2019.28.Sup10.S13 31600101

[13] D. Reifs Jiménez , L. Casanova‐Lozano , S. Grau‐Carrión , and R. Reig‐Bolaño , “Artificial Intelligence Methods for Diagnostic and Decision‐Making Assistance in Chronic Wounds: A Systematic Review,” Journal of Medical Systems 49, no. 1 (2025): 1–39. 10.1007/s10916-025-02153-8 PMC11839728 39969674

[14] A. Stefanelli , S. Zahia , G. Chanel , R. Niri , S. Pichon , and S. Probst , “Developing an AI‐Powered Wound Assessment Tool: A Methodological Approach to Data Collection and Model Optimization,” BMC Medical Informatics and Decision Making 25, no. 1 (2025): 297. 10.1186/s12911-025-03144-y PMC12335118 40783534

[15] S. Freeman , M. J. Sargent , L. R. Galarza , R. McAloney , and E. Rossnagel , “Use of Artificial Intelligence‐Driven Wound Care Management to Enhance Access to Care Rural and Northern Communities,” International Wound Journal 22, no. 10 (2025): e70767. 10.1111/iwj.70767 PMC12457695 40988324

[16] K. Y. Fong , T. P. Lai , K. S. Chan , et al., “Clinical Validation of a Smartphone Application for Automated Wound Measurement in Patients With Venous Leg Ulcers,” International Wound Journal 20, no. 3 (2023): 751–760. 10.1111/iwj.13918 PMC9927911 36787270

[17] T. Dege , B. Glatzel , V. Borst , et al., “Patient‐Centered Chronic Wound Care Mobile Apps: Systematic Identification, Analysis, and Assessment,” JMIR mHealth and uHealth 12 (2024): e51592. 10.2196/51592 PMC11004612 38533818

[18] German Clinical Trials Register (DRKS) . “Segmentation and Progress Monitoring of Chronic Wounds Using Artificial Intelligence (WUNDERKINT): Pilot study. DRKS00038408. Registered 2025‐11‐13,” https://drks.de/search/en/trial/DRKS00038408/.

[19] R. R. Selvaraju , M. Cogswell , A. Das , R. Vedantam , D. Parikh , and D. Batra , Grad‐Cam: Visual Explanations From Deep Networks via Gradient‐Based Localization. In: Proceedings of the Proceedings of the IEEE International Conference on Computer Vision.

