# Supplementary material for: AI‐Based Wound Measurement in a Telemedicine‐Style Workflow: Agreement With In‐Person Reference Measurements Compared With Individual Physicians and Expert Consensus
Source: Int Wound J. 2026 Oct 15;23(10):e71043. doi: 10.1111/iwj.71043 (PMC13673234; doi:10.1111/iwj.71043)
Supplement: Supplementary file 1 [file IWJ-23-e71043-s002.zip › supplemental_material_1.pdf]

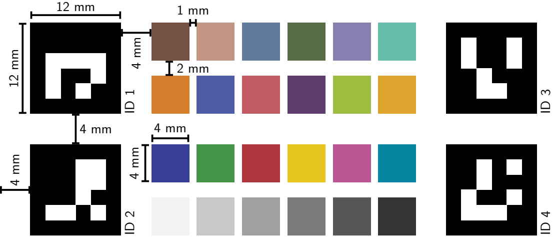
12 mm
1 mm
12 mm
4 mm
2 mm
ID 1
ID 3
4 mm
4 mm
4 mm
4 mm
ID 2
ID 4
